# Removal of Deposits upon Teeth

**Published:** 1897-05

**Authors:** I. A. Freeman

**Affiliations:** Chicago


### Removal of Deposits Upon Teeth.

Read before the Chicago Dental Society, March 2, 1897.

BY I. A. FREEMAN, D.D.S., OF CHICAGO.

In bringing this subject before the Society it is not the intention to discuss the subject of pyorrhea alveolaris as such, but the removal of deposits which are analagous to it; neither is it intended to treat of the different kinds of calcific concretions, or the causes of these deposits found upon the teeth to a great extent. Deposits are defined as "that which is deposited, thrown, or laid down, especially matter precipitated from a solution in water or any other liquid."

Various authors give the origin of the calcific deposits found upon the teeth as being the saliva and that of the blood serum. Climatic influences govern, to some extent, the amount and rapidity with which this deposit is made. In a warm climate, with dry atmosphere, there seems to be far less accumulation than is found upon the teeth of people inhabiting marshy and damp regions, and who, as a result, are troubled with malarial affections. Those persons who are poor eliminators of the waste products of the system are more subject to these concretions than those who are known as good eliminators. Age has its influence. For instance, when the osseous formation is completed there seems to be less demand for the lime assimilated from the different foods, and so the secretions become loaded with it. Now, with poor elimination, the presence of carbonic acid gas as waste in the secretions gives to them the solvent properties, so they are loaded with lime-salts, and, as a consequence, in different cavities of the body are found these products—stone in the bladder, gall-stones, calcific deposits upon the teeth, etc.

Pathological conditions have a varying influence upon the amount and the rapidity of these accumulations. That which is the more rapidly deposited is, as a rule, soft and of light color, while that which is less rapidly laid down is very hard and dark in color. Where there is a rapid development of calcific concretion the sufferer will usually find it necessary to seek a dentist for relief frequently, thus removing one of the causes of the dark, hard variety, which is said to be the cause of the action of lime, the solidity or hardness and color being the result of long-standing habits, etc., which, not being deposited rapidly, does not irritate the gums, and so does not drive the patient to the dentist on that account.

Now there are several means suggested for the removal of these deposits. If a solvent could be found that would not be injurious to the tooth-substance, and yet be sufficiently powerful to cause the softening of this formation known as salivary calculus, it would, indeed, be a great boon to the people. It is said that carbonic acid is a good solvent of lime. Possibly carbonized waters, as furnished for soda fountains, might be found beneficial as a wash for the mouth. Perhaps, by constant use, it would lessen the accumulation, but might not prove to be so helpful as some other kind of acid. Aromatic sulphuric acid is favored by some, both as a stimulant in pyorrhea pockets and also as a solvent of lime which may be left after surgical interference.

Dr. Harlan has suggested trichloracetic acid in dilute form, as being both safe and helpful as a stimulant, and also as a solvent agent, to be used after removal of the deposit by instrumentation.

At a meeting some time since Dr. Younger said he used the commercial strength of lactic acid for the purpose of dissolving the small particles of serumal calcific deposits that may have escaped the instrument. Iodin has been suggested and used as a solvent of these deposits, especially that of green stain found upon the teeth of children. Iodin in other forms has been used with electricity, the theory being, that by the decomposition of iodin the electrolytic action was also had upon the lime-salts present in the nodules or scales upon the teeth. I must confess that I have had but a very limited experience with these agents, and I depend mainly upon surgical interference in my practice, using instruments such as are found in our dental depots in great variety. And here I desire to say a word concerning the instruments provided for this purpose, comparing them with those found in the dental depots thirty years ago, for at that time the instruments were crude, heavy and totally unfit for the purpose intended. It is said that Riggs' disease, not yet known as pyorrhea alveolaris, was incurable, and I believe it was rarely ever treated, as with those instruments it was impossible to remove deposits situated below the gums without nearly murdering the patient; while to-day we are provided with beautifully-constructed instruments, and it is quite possible to reach and remove the scale or nodule, though it be situated well-nigh at the apical extremity of the tooth. Furthermore, we had not at that time another agent which is so helpful and so full of relief to the patient, cocain. Surely along this line dentistry has made one of its greatest advances. The set of instruments suggested by and known as Dr. Cushing's set of scalers or cleansers, with those forms added by Dr. Harlan, as now manufactured, are the most ideal of any, although there are many forms suggested by others that are very helpful and important adjuncts to those mentioned, and I presume each dentist has some forms of instruments of his own device which he regards as indispensable in his practice and are to him the great desiderata.

We come now to consider briefly some of the preliminary steps in removing deposits upon the teeth. I think it will always be best to first take observations, that we may come to a knowledge of all the conditions—what kind of a deposit we have to deal with; whether it be that which is rapidly deposited, light in color, soft and quite easily removed; or of the dark, hard variety; for if of the former the time necessary for its removal will usually be less than if it be of the latter. Then the form of the teeth should be taken into consideration, whether large, bell-shaped crowns, or short, small, and of conical form; whether irregular or well arranged in the dental arch; if in close contact; if the denture be complete; if there be considerable recession of the gum, for the extent of this lesion will have a very considerable influence on the facility with which the operation may be done. These are some of the points we shall do well to observe, for there will be scarcely any limit to the degree of thoroughness demanded in the work to be undertaken.

The operator should always first become acquainted with the case he has in hand and prepare himself for the accomplishment of the work desired. I think it best that the patient be informed with regard to the importance of the operation and the amount of labor necessary for its accomplishment, so that the operator may feel free to make such reservation of time, either for a continuous sitting or for subsequent sittings, as will result in perfect work. I think that the time and physical exertion consumed in properly cleaning a set of teeth which are affected to any considerable extent or have any amount of deposit upon them, and the subsequent treatment of the gums and teeth, are least appreciated in importance by the dentist himself, and, as a consequence, by his patient, of any operations that we, as dentists, are called upon to perform.

I fear that we have not, as a rule, been thorough enough, or have not used anything like the time we should to accomplish the object sought, namely—the cure and eradication of the disease or trouble. Doubtless one reason why some dentists are always busy and have their time fully engaged in advance is because of their exhaustive painstaking in all operations submitted to them. The fact is, we should never be in a hurry about our work. If our patients can not give the time necessary to complete an operation at one sitting, I believe we should arrange for and do only such a part as can be done perfectly in the time set apart for them. For instance, if you can get your patient to sit for only such a time as will be required to clean one tooth in the most thorough manner, why do that and no more; then proceed with the surfaces adjacent or next to the one so completed, but do not leave the first until you have done with it. It may be urged that hemorrhage from wounded and bleeding tissues will cover up the deposit to be removed, so that the work will be greatly hindered if confined to one locality. In reply to this I would say that the smaller the area so wounded the less will be the hemorrhage and obstruction, and then with proper preparation we can control it to a large extent.

And here we come to consider the preparations for the performance of this work. First, the patient seated in the operating chair should be carefully protected, by placing towels about the neck and lap, from having the clothes soiled. Instruments should be sterilized and so placed that they will be within easy reach, and should be of sufficient variety to reach all surfaces as readily as possible, whether regular or irregular, in close contact or well separated. These instruments should be of metal throughout; wood and ivory handles should be discarded. There should be at hand a large syringe for the purpose of washing away the disloged particles, and care should be taken that all calcific spiculæ be removed, for if left within or in contact with the soft tissues they will become a source of irritation. Rolls and pellets of absorbent cotton and folds of bibulous paper should be ready with which to absorb secretions consequent upon the disturbance of the tissues and excitation of the salivary glands.

A mouth-mirror, which has been warmed by either a bath in warm water or by dry heat, so that condensation of moisture from the breath upon it may be avoided, and two or three silver probes of different forms and sizes should be at hand, to be used as explorers of the surfaces beneath the free margin of the gums, by the delicate manipulation of which one may become an adept in the use of them, so as almost to see by them. Indeed, as in other departments of surgery, they are the eyes of the operator to discover the hidden nodules or scales, roughened surfaces and ragged alveolar borders. Now for this delicacy of touch so necessary for the detection of these minute particles of calcific deposits the fingers must be well cared for; should not be roughened by manual labor or toughened by the handling of hot metals or the hot end a cigaret, or otherwise made incapable of that sensitiveness necessary to reveal the most slightly roughened surface upon the teeth or the parts the probe may come in contact with, for if we expect to succeed in our endeavor to cure the case we have in hand we must discover and remove every particle of foreign substance, concretion or deposit.

But there is another instrument needed to be used in treating the parts before we can even approach, in many instances, the hidden offending nodule, so great is the pain caused by forcing away the gums, swollen and sensitive as we often find them in pyorrhea cases. These tissues must be anesthetized before operating upon them, a syringe of delicate construction to carry a mild solution of cocaine, preferably 2 to 4 percent, well down into these pockets, should be at hand. Folds of bibulous paper should be placed around the parts, so as to take up the excess which may escape from the pocket, and thus prevent its absorption by other parts or tissues. With these preparations made, we are ready to proceed, having selected the tooth upon which to begin, which will, of course, depend upon what the conditions are and the extent to which the lesion has developed. The instruments which reach the exposed parts most readily will be required first. For instance, if working upon the lower incisors a long, curved instrument, used by exerting force toward the gum margin along the lingual side, is the form of instrument usually preferred, the little finger resting upon the cutting edge of tooth, thus guarding against undue injury should the deposit be suddenly dislodged.

The mallet may here be used when the force required to dislodge the scale is so great that it is unsafe to use hand pressure alone, which sometimes happens when the dark, hard variety is to be removed. When the surface has been cleared, other very thin instruments, sharpened to an edge, may be pushed through from the labial aspect, and in this way the lateral sides may be cleaned. To supplement these a broad, thin, hatchet-shaped instrument may be used with good results from the lingual aspect. When the lingual surface needs treatment a straight, thin instrument, with force exerted from the cutting edge toward the cervix is best. These instruments will be found sufficient in an uncomplicated case, but should the deposit be beneath the gum, upon the labial or lingual surface, it will be necessary to have other forms and more delicate instruments. These are found in the set advised by Dr. Cushing.

For the removal of deposits situated beneath the gum margin and extending to the apex, instruments that form a short right angle to the shaft will be found best adapted, and they are to be used by carrying past the nodule, then by pressure toward and upon the root, and traction made toward the cutting edge, the right angle end or edge catching up and removing some portion of the deposit with each motion or traction. By repeated and persistent efforts the deposit may be removed and the rough surface smoothed. Of course other instruments will be found helpful; sometimes the scale may be removed entire by the long, thin, curved instrument suggested by Dr. Harlan, the force being exerted toward the apex.

After the various surfaces have been cleared of the large mass of calcific matter, finely pulverized pumice-stone should be applied by means of rubber cup-discs, moose-hide points, wood points, etc. Brushes are helpful and have their place. Along the root, beneath the gum, a piece of soft pine or white wood will carry the powder, and by rubbing carefully will sufficiently smooth those surfaces. Frequently during and after this procedure the syringe should be used with a mixture of some antiseptic in warm water, preferably Black's 1, 2, 3, ℥i; alcohol, ℥iii; glycerine, ℥ss. Use ʒi in one glass of water, and it makes a very pleasant wash for the patient to use. The syringe should be one upon which sufficient force can be applied to wash away the powder, debris, coagulated blood, etc.

This constitutes the typical procedure and will sufficiently indicate the treatment of this lesion, except where a surgical operation is indicated. Of course, there are difficulties attending the treatment of each individual tooth, for which special instruments will be needed to be able to reach all surfaces, and the ingenuity of the operator will be brought into play to overcome them, but he must be patient and persistent, especially with the molars, where each root must be treated as an individual tooth, and where the advantage of everybody's effort to produce an instrument for rendering each difficult operation as simple as possible will be duly appreciated.

DISCUSSION.

Dr. F. R. Ross: Only one thing more occurs to me—that we must remember we are working on human tissue and that it is very painful. I can speak from personal experience, for I was in the chair at a clinic once for three hours, and the on-looking dentists pulled and hauled me around as if I were insensible to pain.

Dr. G. V. Black: This is a matter of great importance, the greatest in dentistry. I see more teeth lost from this disease than from caries, and people come continually with the peridental membrane permanently injured by these deposits. The first thing to do is to remove the deposits with the instruments described. Practice handling until you know them, and where to place them against the roots of teeth to remove deposits. The importance of skill in this work is not yet appreciated by the dental profession, and the same may be said of the treatment. Cases come with the peridental membrane badly destroyed, and yet are dismissed after a little scraping. I have had patients present with half the membrane around the affected teeth gone, and yet they have used the teeth to chew with for ten or fifteen years afterward. The teeth did not grow worse, nor did the membrane. Such patients were educated to come for treatment once in from two to six months, and it was a lifelong task, but it was possible to keep up the use of the teeth, so that the food could be masticated with a degree of comfort. Teeth in this condition should be used as vigorously as possible in order to keep them and preserve their health.

Dr. Garrett Newkirk: I would simply add emphasis to the sterilization of instruments. It is most important here, as a wound with these instruments is very apt to infect the soft tissues. Scalers with hooks are difficult to keep clean, but there is no means so certain for sterilization as boiling after use and leaving with some preparation, such as oil of cassia, on them.

One case occurs to me, where the lower incisors were badly loosened by pyorrhea and crowded, and one was in a hopeless condition. The worst one was removed and the others were cleaned and then drawn together by ligatures, apparently curing the case. Where the teeth were so crowded that one could be spared, and a condition of drainage established, cases have repeatedly been cured by removal of the worst one.

Dr. E. R. Carpenter: There is one point—the necessity of working on but one tooth at a time. In removing these deposits take a single tooth, select the necessary instruments, then diagnose carefully, so that you may know how to proceed after you begin the work. I cocainize the gum with a 10-percent solution, covering the surrounding gum with cotton, and never leave a tooth until I think it is perfectly clean.

Dr. E. Mawhinney: I have not been able to use cocaine enough at one sitting to do much good, so for two years I have used a 2-percent solution of trichloracetic acid before removing these deposits. Dry the gum, wrap cotton around a thin scaler and dip in solution, then carry cotton all around deposit and leave it there for a moment. The astringent action prevents hemorrhage, and the acid has all the deadening effect of cocaine.

Dr. C. P. Pruyn: It is impossible to do much without thorough education of the patients. Where the deposit has not progressed to annoyance, unless the gravity of the situation is impressed upon them, they think you are too cautious, or are trying to make work. When patients are given to understand what the results will be if the deposits are not removed, some success may be attained. I think the essayist's solution of cocaine is not strong enough. When used locally a 10 to 20-percent solution is not dangerous and gives better results.

Dr. Don M. Gallie: In mouths where the teeth are surrounded by calcareous deposits, we more frequently find the inferior incisors injured by the deposits than any other teeth in the mouth. I recall a case that came under my care over five years ago, where the lower incisors were so loose from deposits that there was danger of removing the teeth by using the ordinary methods of scaling. To guard against this I took a strip of modeling composition and took a shallow impression of the anterior teeth, allowed it to harden in ice-water, then trimmed the strip labio-lingually until it was about one-quarter of an inch thick, and placed it back upon the cutting edges of the teeth, thus holding them all firmly in place. It was then an easy matter to remove the immense deposits that were present, and after scaling as thoroughly as possible I ligatured the teeth together, allowing the strip to remain until they were all firmly ligatured. I used the strip at each subsequent sitting. About three weeks ago I saw the patient for the first time since the operation was completed, and was pleased to see the teeth firmly attached and the gum considerably restored, owing mainly, in my opinion, to the method employed.

Dr. C. F. Hartt: One theory advanced to account for the formation of calcareous deposits is, that the lime-salts in the saliva are precipitated by the carbonic acid in the breath, and so form on the teeth. It would, therefore, seem inadvisable to prescribe carbonized waters, as the essayist has suggested.

---

Keep in, to the most pronounce degree, the procession of the medical profession, keep in the front of the procession, in the band wagon if you can, if not, somewhere along the line even at the tail end, don't drop out—the doctor who drops out of the procession, or hesitates, is lost—in other words, make up your mind to keep ahead of the times rather than behind them. —*Love's Lance.*

This is for the dentist as well.—Ed. Reg.

